# Factors Influencing Recombinant Protein Secretion Efficiency in Gram-Positive Bacteria: Signal Peptide and Beyond

**DOI:** 10.3389/fbioe.2019.00139

**Published:** 2019-06-11

**Authors:** Chong Peng, Chaoshuo Shi, Xue Cao, Yu Li, Fufeng Liu, Fuping Lu

**Affiliations:** ^1^Key Laboratory of Industrial Fermentation Microbiology, Education Ministry of China, Tianjin, China; ^2^National Engineering Laboratory for Industrial Enzymes, Tianjin, China; ^3^Tianjin Engineering Research Center of Microbial Metabolism and Fermentation Process Control, Tianjin, China; ^4^College of Biotechnology, Tianjin University of Science and Technology, Tianjin, China

**Keywords:** signal peptide, recombinant protein, secretory pathway, gram-positive bacteria, secretion efficiency

## Abstract

Signal peptides are short peptides directing newly synthesized proteins toward the secretory pathway. These N-terminal signal sequences are ubiquitous to all prokaryotes and eukaryotes. Signal peptides play a significant role in recombinant protein production. Previous studies have demonstrated that the secretion amount of a given target protein varies significantly depending on the signal peptide that is fused to the protein. Signal peptide selection and signal peptide modification are the two main methods for the optimization of a recombinant protein secretion. However, the highly efficient signal peptide for a target protein with a specific bacterial expression host is not predictable so far. In this article, we collect several signal peptides that have previously performed well for recombinant protein secretion in gram-positive bacteria. We also discuss several factors influencing recombinant protein secretion efficiency in gram-positive bacteria. Signal peptides with a higher charge/length ratio in n-region, more consensus residues at the−3 and−1positions in c-region and a much higher proportion of coils are more likely to perform well in the secretion of recombinant proteins. These summaries can be utilized to the selection and directed modification of signal peptides for a given recombinant protein.

## Introduction

In both eukaryotic and prokaryotic cells, all proteins are synthesized in cytoplasm. Proteins that are destined to enter into the secretory pathway are usually endowed with an N-terminal signal sequence: the signal peptide (SP). SPs are short peptides and usually have a length of 16–30 amino acids. After directing proteins to their specific locations, SPs are removed by signal peptidases (Blobel and Dobberstein, 1975; von Heijne, 1990, 1998; Molhoj and Dal Degan, 2004). Research on SPs is quite appealing in the field of protein secretion mechanism. Additionally, research about SPs is valuable in medical research such as disease diagnosis and treatment. For example, mutation in the preproinsulin signal peptide is associated with the onset of diabetes (Bonfanti et al., 2009). A new identified variant in SP of the human luteinizing hormone receptor (LHCGR) affects receptor biogenesis and would cause Leydig cell hypoplasia (Vezzoli et al., 2015). Jarjanazi et al. (2008) carried out a comprehensive literature survey and retrieved 26 disease associated mutations in the signal peptide domains of 21 human proteins (Jarjanazi et al., 2008).

Signal peptides also play a decisive role in the industrial production of recombinant proteins. There is a tremendously strong market demand for recombinant proteins such as industrial enzymes and biopharmaceutical proteins (Walsh, 2018). Different prokaryotic and eukaryotic expression systems have been developed to produce recombinant proteins. Among them, bacterial systems are most attractive because they are simple to manipulate and cost-effective (Terpe, 2006). However, the accumulation of recombinant proteins in the cytoplasm will lead to the formation of inclusion bodies or protein degradation via proteases (Mergulhao et al., 2005; Anne et al., 2016). The recombinant protein folding may also be disturbed by endogenous proteins. If the recombinant protein is secreted out of the cell, the above bottlenecks in the mass production of recombinant proteins can be avoided, and the downstream recovery process of protein production will also be considerably simplified. Thus, developing an efficient secretion system will contribute a lot in the high yield of recombinant proteins (Quax, 1997). It has been shown that using different homologous or heterologous signal peptides can affect the yields of recombinant proteins (Degering et al., 2010; Low et al., 2013; Hemmerich et al., 2016; Kleiner-Grote et al., 2018). Selecting a proper signal peptide to increase the secretion efficiency becomes a common methodology to optimize the production of recombinant protein.

**Figure 1:**
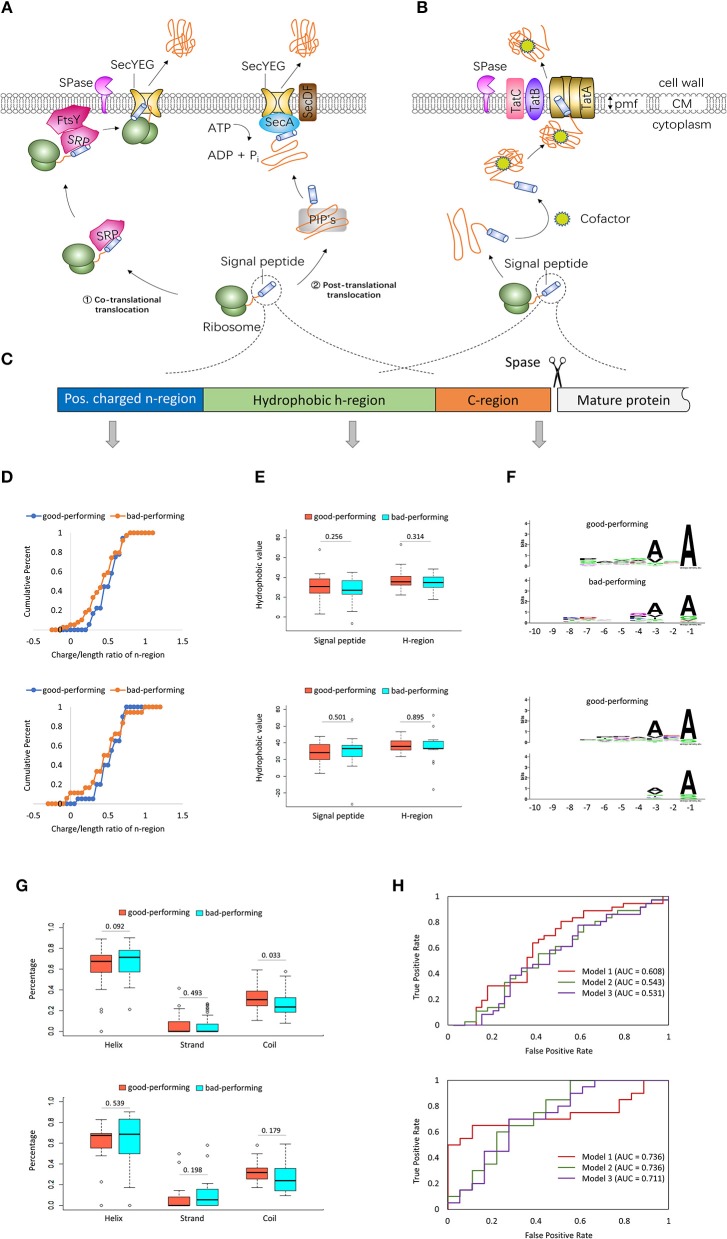
Two major gram-positive bacterial export pathways and signal peptides with different secretion efficiencies. **(A)** The general secretion (Sec) protein export pathway in gram-positive bacteria. (1). In the co-translational export mode, preproteins are recognized at the ribosome by the signal recognition particle (SRP). Then the SRP membrane receptor FtsY binds to the ribosome-nascent chain (RNC)-SRP complex. SRP and FtsY target the preproteins to the transmembrane SecYEG channel. (2). In the post-translational export mode, precursor proteins are fully synthesized and are kept in an unfolded secretion-competent state by the post-translationally interacting proteins (PIP's), such as the general chaperones GroELS/DnaK-DnaJ-GrpE/trigger factor, the CsaA protein and the soluble form of SecA. Then the motor protein SecA translocates the preproteins through SecYEG using metabolic energy from ATP hydrolysis. SecDF enhances the release of preproteins. **(B)** The twin-arginine translocation (Tat) export pathway in Gram-positive bacteria. After being synthesized, the Tat-dependent pre-protein folds rapidly into its native conformation, sometimes with the help of cofactors. The energy for translocation comes from the proton motive force (PMF). In gram-positive bacteria with high GC-content genomes, the Tat translocase consists of TatA, TatB, and TatC. In low-GC gram-positive bacteria, the Tat system is composed of TatC and a bifunctional TatA protein. **(C)** The general structure of signal peptides. Adapted by permission from Springer Nature Customer Service Center GmbH: Springer Nature, Nature Biotechnology (Molhoj and Dal Degan, 2004), copyright 2004. **(D)** Cumulative distributions of the charge/length ratio of n-region in good-performing and bad-performing signal peptides. **(E)** Boxplots of the total hydrophobic values of signal peptides and the hydrophobic values in h-regions. **(F)** Sequence logos of c-region aligned by their cleavage sites in good-performing and bad-performing signal peptides. **(G)** Boxplots of the proportions of helices, strands, and coils in good-performing and bad-performing signal peptides. **(H)** ROC curves of models trained with 1 parameter (dark red), 26 parameters (dark green), and 29 parameters (purple). The data used in the upper half of **(D–H)** are from Brockmeier et al.'s study (2006). Good-performing SPs are the top 36 SPs showing high cutinase activity (top 25% of all SPs). Bad-performing SPs are the 39 SPs showing no cutinase activity (the lower 27% of all SPs). The data used in the bottom half of **(D–H)** are from Zhang et al.'s study (2016). For the 114 Sec-type signal peptides with promoter P43, the top 20 SPs with Xylanase activity > 100 units/ml are selected as good-performing signal peptides. The last 18 SPs with Xylanase activity < 1 units/ml are selected as bad-performing signal peptides.

Gram-positive bacteria usually consist of only one cell membrane. The secretion of a target protein in gram-positive bacteria is thought to be more efficient (Freudl, 2013; Anne et al., 2016). Various gram-positive bacteria, especially the generally recognized as safe (GRAS) gram-positive model bacterium *Bacillus subtilis* (Sewalt et al., 2016), are widely utilized for expression of recombinant proteins in biotechnology (Sone et al., 2015; Anne et al., 2016; Freudl, 2018). Several different protein export systems have been identified in gram-positive bacteria to date, including the general secretion (Sec) pathway, the twin-arginine translocation (Tat) pathway and type VII/WXG100 secretion systems. Figures 1A,B are the schematic figures of Sec and Tat export pathways in gram-positive bacteria. Sec-dependent proteins are translocated to the plasma membrane either co- or post-translationally (Figure 1A). In the co-translational export mode, precursor proteins are recognized at the ribosome by the signal recognition particle (SRP) and then targeted to the transmembrane SecYEG channel by SRP and FtsY, the SRP membrane receptor (Elvekrog and Walter, 2015). In the post-translational export mode, the post-translationally interacting proteins (PIP's), such as the general chaperones GroELS, DnaK-DnaJ-GrpE, trigger factor, the CsaA protein and the soluble form of SecA, keep the fully synthesized precursor proteins in an unfolded secretion-competent state (Wu et al., 1998; Herbort et al., 1999). Then the motor protein SecA translocates the preproteins through SecYEG using metabolic energy from ATP hydrolysis (Schiebel et al., 1991). In addition, SecDF enhances the release of preproteins (Tsukazaki et al., 2011). Tat-dependent proteins are transported across lipid bilayers in a folded state (Figure 1B). The energy for translocation comes from the proton motive force (PMF). In gram-positive bacteria with high GC-content genomes, the Tat translocase consists of TatA, TatB, and TatC. In low-GC gram-positive bacteria, the Tat system is composed of TatC and a bifunctional TatA protein (Goosens et al., 2014). These two and other different types of secretion machinery have been well-reviewed in several excellent articles (Palmer and Berks, 2012; Freudl, 2013; Goosens et al., 2014; Ates et al., 2016; Green and Mecsas, 2016; Tsirigotaki et al., 2017; Owji et al., 2018). Readers can refer to these reviews for a better understanding of the protein secretory mechanisms in gram-positive bacteria.

Based on the export pathways of the preproteins and the signal peptidase cleavage sites, signal peptides can be classified into several categories, among which Sec-type signal peptides and twin-arginine signal peptides are more abundant and well-studied (Tjalsma et al., 2000, 2004). Signal peptides from different proteins show a common structure. Generally, a signal peptide is composed of three distinct domains: a positively charged n-region (1–5 residues long), a central, hydrophobic h-region (7–15 residues long), and a c-region (3–7 residues) with the cleavage site of signal peptidase (von Heijne, 1985, 1990). The general structure of signal peptides is shown in Figure 1C. A highly conserved twin-arginine motif (SRRXFLK, where X is often, but not always, a polar amino acid residue) is located at the n/h-region boundary of Tat-specific signal peptides (Berks, 1996; Berks et al., 2000). Several bioinformatic tools have been built and maintained by different research groups to predict signal peptides, such as SignalP (Petersen et al., 2011), Phobius (Kaell et al., 2007), PrediSi (Hiller et al., 2004) for Sec-type signal peptides and TatP (Bendtsen et al., 2005), Tatfind Server (Rose et al., 2002), PRED-TAT (Bagos et al., 2010) for twin-arginine signal peptides (Caccia et al., 2013).

This article is a brief review of factors that influence signal peptide secretion efficiency for recombinant protein in gram-positive bacteria, especially in *B. subtilis*. We summarize several experimental achievements in the screening of a proper signal peptide for a given protein. We also discuss the differences between good-performing and bad-performing signal peptides for different recombinant proteins in *B. subtilis*. Additionally, other factors including the pro-region of recombinant protein and the expression host are also summarized in the last part.

## Optimization of Recombination Protein Secretion by Signal Peptide Screening

Generating a signal peptide library has proven to be a practicable approach for the optimal secretion of recombinant proteins in Gram-positive expression hosts. The first effort to systematically search the best-performing signal peptide for heterologous protein secretion was performed a decade ago. In this study, a signal peptide library consisting of 173 predicted Sec-type SPs from *B. subtilis* strain 168 was constructed (Brockmeier et al., 2006). The functionality of each SP was studied using cutinase from *Fusarium solani pisi* as the reporter protein. *B. subtilis* TEB1030 was used to express the SP-cutinase fusions. This study reveals that the enzymatic activities of cutinase vary significantly when different SPs were fused to the protein. A similar conclusion was also obtained in the comprehensive analysis of signal peptide functionality from *Lactobacillus plantarum*. Mathiesen et al. (2009) constructed a library of 76 Sec-type signal peptides from *L. plantarum* WCFS1. Staphylococcal nuclease (NucA) was used as the reporter protein. This screening showed considerable variation in the levels of secreted NucA (Mathiesen et al., 2009). In another experiment, 405 candidate signal peptides were predicted in the completely sequenced genome of *Corynebacterium glutamicum* R. Then each of the SPs was fused to a heterologous α-amylase (AmyE) from *Geobacillus stearothermophilus*. A total of 108 SPs were shown to mediate detectable secretion of AmyE from the expression host *C. glutamicum* R. Eleven of these samples exhibited 50- to 150-fold higher secretion level than that of the signal peptide derived from the well-known corynebacterial secretory protein PS2 (Watanabe et al., 2009).

A promoter is defined as the region of DNA sequence that initiates the gene transcription (Wrighton, 2018). Promoters are often used together with signal peptides as regulatory elements for the expression and production of recombinant proteins (Guan et al., 2016; Gu et al., 2017; Maffei et al., 2017; Cui et al., 2018). Zhang et al. (2016) performed an experimental screen of 138 signal peptides from *B. subtilis* for the production of an alkali-tolerant xylanase (XynBYG) from *Bacillus pumilus* BYG. They used *B. subtilis* WB700 as the expression host. Two promoters (P*glvm* and the constitutive promoter P43) were separately used in the expression of the protein. The yields of XynBYG using P*glvm* promoter were higher than using the P43 promoter, which indicated that P*glvm* promoter is more efficient than the P43 promoter for XynBYG expression. In further analysis, an obvious correlation with a Pearson correlation coefficient of 0.97 was observed between the yields of XynBYG driven by the two promoters. In other words, good-performing SPs would have higher secretion efficiency than bad-performing SPs no matter which promoter is used in the expression of the protein, and vice versa. This work indicates that promoters do not affect the secretion performance of signal peptides. If a signal peptide performs well when using promoter A in the expression of the target protein, it will also perform well when promoter B is used (Zhang et al., 2016).

Signal peptide library construction followed by high-through screening has also been reported in the secretion of several recombinant proteins (Degering et al., 2010; Tsuji et al., 2015; Cai et al., 2016; Hemmerich et al., 2016). Featured with high efficiency and high coverage of SPs, this method has screened many good-performing signal peptides for different recombinant proteins. Table 1 shows the signal peptides that have previously performed well in gram-positive bacteria. Apart from the signal peptide library-based method, there are also plenty of researches, too numerous to be entirely listed, in which a few signal peptides are involved (Freudl, 2018; Kalbarczyk et al., 2018; Owji et al., 2018). If the secretion efficiencies of these SP-protein combinations are gathered up in specific database, they will be of great value for signal peptide selection and further data analysis.

**Table 1:** Examples of several signal peptides that perform well in gram-positive bacteria.

**Signal peptide**		**Recombinant protein**		**Host**	**Yield**	**Ranking**	**Reference**
**Signal sequence**	**Origin**	**Protein**	**Origin**				
MKNMSCKLVVSVT LFFSFLTIGPLAHA	*B. subtilis, Epr*	Cutinase	*F. solani pisi*	*B. subtilis* TEB1030	4.67 [U/mL]	1/173	Brockmeier et al., 2006
MAKPLSKGGILVKKVLIAGA VGTAVLFGTLSSGIPGLPAADAQVAKA	*B. subtilis, YncM*	Aminopeptidase	*B. subtilis* Zj016	*B. subtilis* WB600	88.59 [U/mL]	1/20	Guan et al., 2016
MKKFNFKTMLLLVLASCVFGVV VNVTTSLGPQTAITAQA	*L. plantarum*WCFS1	NucA (nuclease)	*S. aureus*	*L. plantarum* WCFS1	35.84 [U/mL]	1/78	Mathiesen et al., 2009
MKEVRFWGLLLGL FVCLGAVIPLVSKA	*L. plantarum*WCFS1	AmyA (amylase)	*L. amylovorus* NRRL B-4549	*L. plantarum* WCFS1	3.4 [10^2^mU/mL]	1/18	Mathiesen et al., 2009
MQINRRGFLKA TAGLATIGAASMFMPKANA	*C. glutamicum* R	AmyE (α-amylase)	*G. stearothermophilus*	*C. glutamicum*	288.3 [U/mL]	1/31	Watanabe et al., 2009
MRSKKLWISLLF ALTLIFTMAFSNMSA	*B. licheniformis* WX-02, *AprE*	Nattokinase	*B. subtilis natto*	*B. licheniformis* Δ0F-3	31.99 [FU/mL]	1/81	Cai et al., 2016
MKNMSCKLVVSVTL FFSFLTIGPLAHA	*B. subtilis, Epr*	Cutinase	*F. solani pisi*	*C. glutamicum*	13.1 [U/mL]	1/64	Hemmerich et al., 2016
MKKFPKKLLPIAVL SSIAFSSLASGSVPEASA	*B. subtilis, PhoB*	XynBYG (alkaline active xylanase)	*B. pumilus* BYG	*B. subtilis* WB700	327.2 [U/mL]	1/138	Zhang et al., 2016
MRSKKLWISLLFAL TLIFTMAFSNMSVQA	*B. subtilis*168, *AprE*	Alkaline protease	*B. alcalophilus* TCCC11004	*B. subtilis* WB600	7574.08 [U/mL]	1/35	Our lab
MRIFKKAVFVIMI SFLIATVNVNTAHA	*B. subtilis* 168, *DacB*	Alkaline protease	*B. alcalophilus* TCCC11004	*B. amyloliquefaciens* 111018	19835.7 [U/mL]	1/86	Our lab

## Primary and Secondary Structure of Signal Peptides With Different Secretion Efficiencies

All researches mentioned in the previous section come to the unanimous conclusion that the secretion levels of recombinant protein differ significantly when different SPs are fused to the protein. In other words, the physicochemical properties of SPs may affect the secretion levels of recombinant proteins. To further explore the factors that determine the secretion efficiency of SPs, biologists would also perform some statistical analysis between the yields of target proteins and signal peptide characters such as lengths, charges, pI values, D-scores from SignalP and so on (Zhang et al., 2016; Fu et al., 2018). In this section, we will try to investigate the differences between good-performing and bad-performing signal peptides by *in silico* analysis of SPs.

The *in silico* analysis are performed with 143 Sec-type signal peptides in Brockmeier et al.'s (2006) study and 114 Sec-type signal peptides in Zhang et al.'s (2016) study. For Brockmeier et al.'s data, the top 36 SPs (25% of all SPs) showing high cutinase activity are selected as good-performing signal peptides. The 39 SPs (27% of all SPs) showing no cutinase activity are selected as bad-performing signal peptides. For the 114 Sec-type signal peptides with promoter P43 reported by Zhang et al., the top 20 SPs (with Xylanase activity > 100 units/ml) are selected as good-performing signal peptides. The last 18 SPs (with Xylanase activity < 1 units/ml) are selected as bad-performing signal peptides. The analysis results are displayed in Figure 1.

Figure 1D shows the cumulative distributions of the charge/length ratio of n-region in good-performing and bad-performing signal peptides. The two panels of Figure 1D reveal that the charge/length ratio of n-region in good-performing SPs is higher than that in bad-performing SPs. Previous studies also proved the importance of positively charged residues in the n-region during the initial step of protein secretion across the membrane. Substitution of positively charged residues with uncharged or negatively charged residues would reduce the protein synthesis rate and transport rate (Inouye et al., 1982; Nesmeyanova et al., 1997). Increasing the positive charge of n-region has been demonstrated to improve secretion efficiency in both gram-positive (Takimura et al., 1997; Ng and Sarkar, 2013) and gram-negative bacteria (Ismail et al., 2011). However, it is notable that the increase in the positive charge is not always favorable. The plots in Figure 1D show the prediction power of the charge/length ratio of n-region can be up to 1, and it might be not helpful when the value is above 1. Other studies have shown that increasing the positive charge in n-region reduced the protein secretion (Ravn et al., 2003; Jonet et al., 2012; Gao et al., 2016). We suspect that positively charged residues in h-region and c-region of SP and the mature protein may lead to the contradictory results.

Figure 1E shows the boxplots of the total hydrophobic values in signal peptides and the hydrophobic values in h-regions. The Kyte-Doolittle hydrophobic scale is used in the current study (Kyte and Doolittle, 1982). The Wilcoxon Rank Sum Test reveals that hydrophobic values show no statistically significant differences between good-performing and bad-performing signal peptides (*P*-values > 0.05). Previous studies showed that interfering in the h-region hydrophobicity has various effects on protein secretion. For example, reducing the hydrophobicity of *Staphylococcus aureus* SP completely abolished the secretion of mature protein (Mordkovich et al., 2015). Increasing the h-region hydrophobicity promoted the secretion of the heavy chain of monoclonal antibody in *Escherichia coli* (Zhou et al., 2016). Substitution of Gly with Cys and Leu in the PhoE SP shifted protein secretion from SecB to SRP-dependent pathway (Adams et al., 2002). It is more likely that the order of residues and the secondary structure they formed in h-region regulate the protein secretion efficiency (Zhang et al., 2016; Han et al., 2017).

We also generate the sequence logos of c-region in good-performing and bad-performing signal peptides with the WebLogo service (Crooks et al., 2004) (Figure 1F). The sequence logos are aligned by their cleavage sites. Data from both Brockmeier et al.'s and Zhang et al.' study show that residues at the−3 and−1positions relative to the signal peptidase cleavage site are more consensus in good-performing SPs than in bad-performing SPs. Alanine residues are more likely to appear at positions−3 and−1 in good-performing signal peptides. Early studies have also shown that the presence of Ala residues at positions−3 and−1 resulted in a considerable improvement in recombinant protein secretion (Ravn et al., 2003; Guan et al., 2015).

Figure 1G shows the boxplots of the proportions of helices, strands and coils in good-performing and bad-performing SPs. The secondary structure of signal peptides are predicted by PSIPRED (Buchan et al., 2013). For Brockmeier et al.'s data (2006), the Wilcoxon Rank Sum Test suggests that good-performing signal peptides have a much higher proportion of coils (the upper half of Figure 1G). However, the *P-* values in Zhang et al.'s data (2016) are not significant enough (the bottom half of Figure 1G). In a recent study, a native Sec-type signal peptide and its modified counterpart were used to secrete *Candida antarctica* Lipase B (CALB) in *E. coli*. The molecular dynamic simulation shows that the native signal peptide contains an alpha-helix structure, whereas the designed one consists only coils and turns. The secondary structure of designed signal peptide creates a more stable interaction with the signal peptidase. Their results showed that the designed signal peptide increased the secretion of CALB (Ghahremanifard et al., 2018).

According to the above analysis, we suspect that the secondary structure is critical to the secretion efficiency of a signal peptide. Coils help to enhance the interaction between signal peptides and signal peptidases. The positive charge of n-region, the hydrophobicity of h-region and the Ala residues at the−3 and−1positions in c-region may exert indirect effects on the secretion efficiency of the signal peptide through their effects on the secondary structure of the signal peptide.

To test if it is possible to predict SPs performance based on the above sequence and structure features, we developed three support vector machine (SVM)-based models for each of the two data sets. The models were implemented with the software toolbox LIBSVM 3.23 (Chang and Lin, 2011). In model 1, only 1 parameter, the charge/length ratio of n-region, was used. In model 2, a total of 26 parameters including the charge/length ratio of n-region, the hydrophobic values in h-region, the length of SP, the length of N/H/C region and the frequencies of 20 amino acids in each SP (20 features) were used. In model 3, the proportions of helices, strands and coils in SP (3 features) together with the 26 feathers in model 2 were used. The ROC curve in 10-fold cross-validation tests for each model is presented in Figure 1H. The AUC scores of the three models are between 0.53 and 0.61 for Brockmeier et al.'s data (2006). For Zhang et al.'s data (2016), the AUC scores are between 0.71 and 0.74. Given the immaturity of these models, it would deserve a try to predict SP performance with machine learning methods if more features and more accurate algorithms are added to the prediction models.

## Other Factors Influencing Protein Secretion Efficiency in Action

The experimental researches of signal peptide screening also show that the secretion efficiency is at least in part dependent on the protein that is secreted. In Brockmeier et al.'s study, a subset of signal peptides in the SPs library was fused to a cytoplasmatic esterase of metagenomic origin. Surprisingly, the best signal peptide for cutinase secretion was inefficient for esterase and *vice versa* (Brockmeier et al., 2006). Similarly, in Mathiesen et al.'s study, lactobacillal amylase (AmyA) was also used as the reporter protein with a selected set of SPs. No correlation was observed between the signal peptide performance with NucA and with AmyA. The secretion efficiency of a given signal peptide is changeable when it is fused to different proteins (Mathiesen et al., 2009). The ~30 residues downstream of the signal sequence, termed the “pro-region,” has also been shown to be critical for protein secretion (Andersson and von Heijne, 1991; Low et al., 2013; Musik et al., 2019). Our suspicion is that the pro-region influences protein secretion efficiency through its intervention to the interaction between the signal peptide and signal peptidase.

Degering et al. (2010) constructed a signal peptide library consisting of 173 signal peptides from *B. subtilis* and 220 signal peptides from *Bacillus licheniformis* to improve the production of subtilisin protease BPN' from *Bacillus amyloliquefaciens* ATCC 23844. Three different *Bacillus* expression strains (*B. subtilis* TEB1030, *B. licheniformis* DSM13/MW3, and *B. licheniformis* strain H402) were used as expression hosts. Both homologous and heterologous signal peptides fused to the target protein can direct protease secretion. Strikingly, the majority of SP-BPN' fusions showed similar relative levels of protease secretion in all three *Bacillus* expression strains (Degering et al., 2010). However, in another study, distantly related organisms are used as expression hosts (Hemmerich et al., 2016). In this research, a signal peptide library consisting of about 150 SPs from low-GC firmicutes *B. subtilis* was constructed. Cutinase from *F. solani pisi* used by Brockmeier et al. (2006) was also selected as the model enzyme. The SP-cutinase fusions were successfully transferred to high-GC actinobacterium *C. glutamicum* ATCC13032 as alternative secretion host. The protein secretion levels with the same SP in Brockmeier et al.'s (2006) study (*B. subtilis* as secretion host) and in this study (*C. glutamicum* as secretion host) were compared. Interestingly, no correlation was observed between the two sets of data. Videlicet, the cutinase secretion levels directed by the same signal peptide differ dramatically with *B. subtilis* and *C. glutamicum* as secretion hosts. The results of the two studies show that the phylogenetic distance of expression hosts may affect the secretion performance of specific SP-protein combinations (Hemmerich et al., 2016).

## Conclusion and Perspectives

Secreting recombinant protein out of the cell can improve the yield and simplify the purification process. A highly efficient signal peptide is of great value in the construction of secretory expression system. Signal peptide library construction followed by high-through screening has been successfully applied in the selecting of appropriate signal peptides for a target protein. This technology and other genetic engineering tools such as CRISPER can be further implemented on bacterial systems for the good-performing SPs selection and recombinant proteins production.

*In silico* analysis of good-performing and bad-performing signal peptides reveals that good-performing signal peptides have a higher charge/length ratio in n-region and more consensus residues (alanine amino acids are preferred) at the−3 and−1positions in c-region. Moreover, good-performing signal peptides have a much higher proportion of coils. Except for the signal peptide properties itself, the pro-region of the target protein and the expression host may also influence the secretion efficiency. We speculate that the interaction between the signal peptide and signal peptidase is critical to the recombinant protein secretion efficiency. The primary and secondary structure, as mentioned above, would most likely influence the secretion efficiency of the signal peptide through their effects on the interaction between the signal peptide and signal peptidase. We hope more experimental data can be generated and more regularities about secretion efficiencies can be summed up by bioinformatic approaches. The bioinformatic databases and concluded laws will become great contributors to the selection and directed modification of signal peptides for a given recombinant protein.

## Data Availability

Publicly available datasets were analyzed in this study. This data can be found here: https://www.sciencedirect.com/science/article/pii/S0022283606009272 and https://link.springer.com/article/10.1007%2Fs00253-016-7615-4.

## Author Contributions

FpL conceived and designed the study. CP performed the study and drafted the manuscript. CS, XC, and YL took part in the data collection. FfL took part in the data analysis. All the authors edited the manuscript and approved the final manuscript.

### Conflict of Interest Statement

The authors declare that the research was conducted in the absence of any commercial or financial relationships that could be construed as a potential conflict of interest.
